# The influence of splenic tissue on the survival and TCD4 and TCD8 lymphocyte rates in rats subjected to fecal peritonitis induction^1^

**DOI:** 10.1590/s0102-865020200100000003

**Published:** 2020-11-23

**Authors:** Andréa Saade Daher Borjaili, Giseli Celestino Nunes, Julia Belizário Silveira, Flávia Heiderich Dall'Orto, Gabriel Souza Lorenzoni, Matheus Eduardo Daher Borjaili, Bianca Prandi Campagnaro, Maressa Cristiane Malini de Lima, Marcela Souza Lima Paulo, Danilo Nagib Salomão Paulo, Tarcizo Afonso Nunes

**Affiliations:** I Fellow PhD degree, Postgraduate Program in Surgical and Ophthalmological Applied Sciences, Medical School, Universidade Federal de Minas Gerais (UFMG), Belo Horizonte-MG, Brazil. Scientific and intellectual contributions to the study, analysis and interpretation of data, manuscript preparation and writing.; II Graduate student, School of Sciences, Santa Casa de Misericórdia de Vitória (EMESCAM), Vitoria-ES, Brazil. Acquisition of data, technical procedures.; III Graduate student, School of Sciences, EMESCAM, Vitoria-ES, Brazil. Technical procedures.; IV Graduate student, School of Sciences, EMESCAM, Vitoria-ES, Brazil. Manuscript preparation.; V PhD, Full, Professor, Laboratory of Translational Physiology and Pharmacology, Pharmaceutical Sciences Graduate Program, Universidade Vila Velha (UVV), Brazil. Flow cytometry protocols, analysis of data.; VI PhD, Associate Professor, School of Sciences, EMESCAM, Vitoria-ES, Brazil. Technical procedures.; VII PhD, Associate Professor, School of Sciences, EMESCAM, Vitoria-ES, Brazil. Intellectual and scientific content of the study, manuscript preparation, critical revision.; VIII PhD, Chairman, Full Professor, Department of Surgery, School of Sciences, EMESCAM, Vitoria-ES, Brazil. Intellectual, scientific, conception and design of the study; critical revision; final approval.; IX PhD, Chairman, Full Professor, Department of Surgery, UFMG, Belo Horizonte-MG, Brazil. Intellectual and scientific content of the study, critical revision, final approval.

**Keywords:** Splenectomy, Spleen, Peritonitis, Immunophenotyping, Survival Rate, Rats

## Abstract

**Purpose:**

Comparing survival rates of rats subjected to spleen procedures after fecal peritonitis induction. Assessing changes in TCD4 and CD8 lymphocyte rates before and after the procedures. Correlating animal survival with CD4 and CD8 lymphocytes.

**Methods:**

Thirty male Wistar rats were distributed into 3 groups of ten: spleen manipulation (SM); total splenectomy (TS); subtotal splenectomy with preservation of the inferior pole (IP). Rats were subjected to surgical procedure depending on the group. Seven days after surgery they underwent induction of peritonitis and survival time was recorded. All animals were subjected to two blood collections: before surgery and 70 days after it for TCD4/TCD8 lymphocyte counting.

**Results:**

Mean survival time was longer in the IP and SM groups and shorter in the TS group; there was significant difference between them. The comparison of the median number of CD4 did not present changes, whereas the comparison of the median number of CD8 decreased in the SM and IP groups. The correlation between the median number of TCD4 and TCD8 lymphocytes and the animals’ survival was not significant.

**Conclusion:**

The maintenance of splenic tissue contributed to increase the survival of rats and there was a change in the number of TCD8 lymphocytes.

## Introduction

Spleen is an organ belonging to the phagocytic mononuclear system; it performs several functions, mainly the defense of the organism^1^. It also works as immune filter, capable of cleaning approximately 4% of the total volume of blood in the body per minute, of producing lymphocytes and monocytes, of making phagocytosis of foreign particles, bacteria, viruses and leucocytes, as well as of processing serum factors such as opsonins, which stimulate phagocytosis^2^. Improvements in the knowledge about the spleen and its functions have influenced the adoption of medical conducts focused on preserving the splenic tissue^3^ ^,^ ^4^.

The idea that spleen removal does not account for complications started being questioned when it was shown that this organ was essential for the performance of several defense mechanisms in the body^5^. In 1952, five children subjected to total splenectomy due to spherocytosis suffered with fulminant sepsis^6^. After the publication of the aforementioned study, the risk of developing post-splenectomy infection has been considered to be the same in children and adults.

The role played by the spleen in the homeostasis of the immunological function consists in storing, transforming and proliferating lymphocytes^7^. The spleen is a secondary lymphoid organ with wide cell diversity and highly partitioned microenvironment. This organ holds cells necessary for immunological response development; they are segregated to specific areas and to regions of antigenic interaction and presentation^8^ ^,^ ^9^.

Cells of the immune system are numerous, 25% of the total are lymphocytes T and 10-15% of all are lymphocytes B^10^. Spleen removal represents the loss of an important site for the allocation and control of lymphoid cells; therefore, it influences their organization and circulation^11^ ^,^ ^12^. Besides, it changes the migration pattern of white cells, mainly of lymphocytes, which causes their accumulation in the peripheral blood^13^.

Studies focused on inducing peritonitis in rats have shown that animals subjected to total splenectomy present higher mortality rates than those who were not splenectomized^14^. Yet, animals subjected to total splenectomy associated with autogenous splenic implant also died^15^. Another study has identified lower phagocytosis ability in rats after total splenectomy than in rats with whole spleen, as well as inflammation process induction^16^. Assumingly, whenever it is necessary performing a splenectomy section, it is also necessary preserving the splenic tissue to avoid sepsis. However, there is controversy about the way to keep the splenic tissue; one of the options is to preserve the lower pole after subtotal splenectomy^17^. Nevertheless, there is also controversy about whether this splenic pole is enough to keep the spleen's functions.

Thus, the aims of the present research were to compare the survival rates of rats subjected to different spleen procedure types after fecal peritonitis induction, to assess whether there are changes in TCD4 and TCD8 lymphocyte counting before and after the spleen procedures and to correlate the survival of animals with fecal peritonitis subjected to spleen procedures to TCD4 and TCD8 lymphocytes.

## Methods

Experimental prospective research approved by the Research Ethics Committee on the Use of Animals of the Sciences Higher School of Santa Casa de Misericórdia in Vitória – EMESCAM (CEUA – protocol n. 001/2015) and by the Ethics Committee of Universidade Federal de Minas Gerais – UFMG (CETEA – protocol n. 275/2015).

In total, 30 young male Wistar rats in the age group 2-3 months presenting body weight of 320g were used in the experiment. The animals were kept in appropriate cages, labeled and stored under conditions recommended for the species: controlled light, temperature and ventilation. They were treated with appropriate feed and water *ad libitum*.

The animals were divided into 3 groups (10 animals, each) depending on the conducted procedure. All animals were weighed, anesthetized with ketamine hydrochloride at a dose of 75mg/kg weight (Vetaset®) associated with xylazine hydrochloride at a dose of 5mg/kg weight (Kensol®) through intraperitoneal route. All of them were subjected to supra and medium umbilical median laparotomy. Spleen procedures were performed based on the research design: Group SM – spleen manipulation; Group TS – total splenectomy after ligation and splenic vascular pedicle section close to the spleen; Group IP – subtotal splenectomy with preservation of the inferior pole, which consists in the ligation and section of vessels that supply the upper and middle portion of the spleen, close to the splenic border, with the aid of 5-0 mononylon, and in spleen section below the connected vessels. The inferior pole of the spleen was kept irrigated by gastrosplenic ligament vessels, without suture or by omentum placement on the bloody edge.

### Immunophenotyping

Spleen influence on lymphocyte populations was assessed in the groups of animals through the immunophenotyping of TCD4 and TCD8 cells in peripheral blood collected at the following times: T1 = seven days before the surgical procedure; T2 = 63^rd^ postsurgical day.

In total, 1mL of blood was collected from the tail of each animal (before and after the surgery) and placed in 3-mL test tubes filled with anticoagulant (ethylenediaminetetraacetic acid - EDTA). The isolation of mononuclear cells (MNC) was carried out after each collection time through density gradient (Ficoll). After isolation, cells were re-suspended to the necessary concentration (10^6^ cells/mL) and centrifuged. This procedure was followed by resuspension in cryotube filled with freezing solution (5% dimethyl sulfoxide - DMSO - and 95% fetal bovine serum). The sample added with freezing solution remained in the freezer overnight at −20°C and was stored at −80°C. After all collection times were over, the samples were subjected to thawing protocol in solution with 20% of fetal bovine serum and *Dulbecco's Modified Eagle Medium* (DMEM) heated to 37°C. The thawed samples were centrifuged, washed twice and re-suspended in Phosphate Buffered Saline (PBS). Samples of mononuclear cells were assessed in flow cytometry apparatus.

Peritonitis induction in rats was carried out 70 days after the surgical procedures by administering the suspension prepared with 2g of fresh feces (of the animals themselves) at the dose 10 mL/Kg weight – this dose is known to be lethal^18^. This suspension was prepared by diluting feces in 17 mL of sodium chloride solution at 0.9% and filtering it in gauze to allow its passage through the needle.

Survival time (in hours) comparison among the groups of animals was carried out through parametric Kruskal-Wallis test; multiple comparisons were conducted through Dunn test. Survival analysis was performed through Kaplan Meier estimator and comparison among groups of animals was performed through Log Rank test. The correlation between animal survival time after peritonitis induction and TCD4 and TCD8 lymphocyte rates was carried out through Spearman's correlation test. The comparison between the amount of TCD4 and TCD8 lymphocytes, before and after the spleen procedures, was performed though non-parametric Wilcoxon test – values were significant at p≤0.05.

## Results

All animals subjected to surgical procedure survived until the moment they were administered with the feces injection in the abdomen. The shortest survival time was 0.2 hours and the longest one was 15.8 hours; mean survival time was 9.1 hours and the standard deviation was 4.1 hours.

Median and mean survival time of animals in group 2 (TS) were 3.83 hours and 4.79 hours, respectively; standard deviation accounted for 85.0% of the mean time, and it highlights high variability. Group 3 (IP) recorded median and mean survival time of 11.35 hours and 11.25 hours, respectively; this outcome points towards low variability. Comparison among the three groups evidenced significant difference among them (p=0.013).

**Figure 1:**
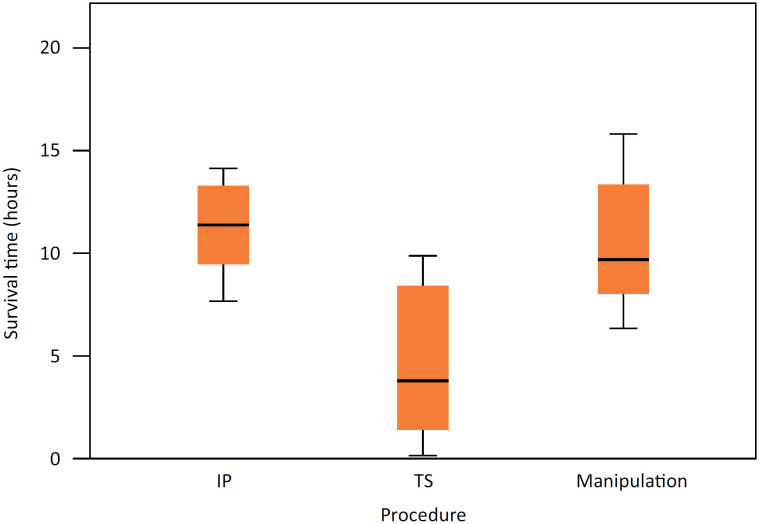
Survival time frame by group of animals.

Note: TS – total splenectomy; STSIP – subtotal splenectomy with preservation of the inferior pole – non-parametric Dunn test IP *vs*. TS – p<0.05 TS *vs*. Manipulation – p<0.05IP *vs*. Manipulation – p>0.05.

The multiple comparisons of survival time among the three groups of animals have shown significant differences among them (Fig. 1).

**Figure 2:**
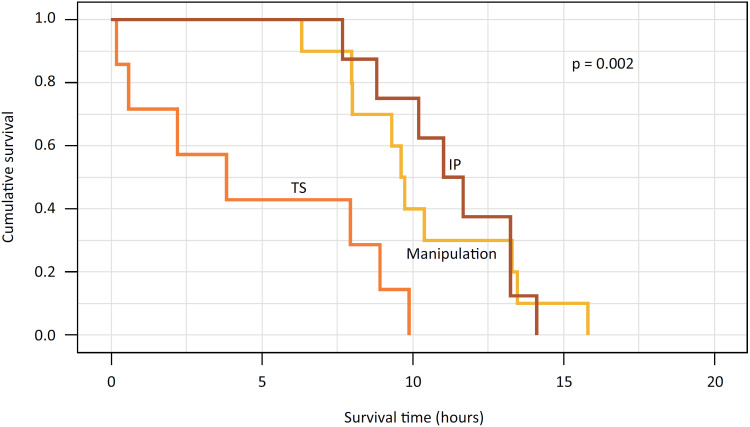
Survival curve of animals per group (Kaplan-Meier). Note: TS = total splenectomy, STSIP = subtotal splenectomy with preservation of the inferior pole.

After peritonitis induction, the chance of survival within 10 hours of animals in group 2 (TS) was of approximately 10%; in group 1 (spleen manipulation), 40%; and in group 3 (IP), 72% - survival curves presented significant difference from each other (p=0.002) (Fig. 2).

The comparison of median number of TCD4 lymphocytes in animals in the three groups, before and after the spleen procedures, did not present difference among them at this stage of the study (p>0.05).

The comparison of median TCD8 lymphocyte counting in animals in the three groups, before and after the spleen procedures, did not evidence differences in group 2 (TS) (p=0.953), but there was difference between two moments in groups 1 (spleen manipulation) (p=0.022) and 2 (IP) (p=0.017).

The number of TCD4 and TCD8 lymphocytes was not correlated to the survival of animals with fecal peritonitis in the three groups (p>0.05).

## Discussion

The research about associations between spleen and infections is justified by controversies about the topic. For a long time, it was consensus that total splenectomy did not lead to severe complications, but it changed when children subjected to such a procedure suffered with fulminant sepsis. Post-splenectomy sepsis is rare, but it is associated with high mortality rate, a fact that justifies the studies about its prevention^19^.

Experimental post-splenectomy sepsis models have reported correlation between these two events, since spleen removal has increased mortality rates associated with exposure to bacteria, and with intranasal and intravenous inoculation. This process is directly related to reduced removal or depuration of these bacteria from the blood stream^20^.

With respect to the present study, peritonitis was induced by feces injections (of the animals themselves) in the abdomen at doses known as lethal. The choice for this model was substantiated by the fact that it provides contamination by mixed flora, similarly to peritonitis in humans, due to several clinical conditions^21^ ^-^ ^23^. The treatment-free peritonitis model led all animals to death within a short period-of-time. This same outcome was reported by other researchers in previous studies, because mortality rates are high at these circumstances, either in the presence of splenic tissue or not^24^ ^-^ ^26^. The option for using Wistar rats resulted from many factors, such as easiness on installing the experiment and on making comparisons to other studies, low cost and easy control and management of these animals in comparison to bigger-sized models^27^ ^-^ ^29^.

Analyzing the survival of rats subjected to different spleen procedure types followed by peritonitis induction was a good way to observe that animals who have undergone total splenectomy had shorter survival time than the ones in the other two groups. Animals in group IP recorded median survival time longer than 11 hours; moreover, this value was the highest among the three groups of animals. This group presented the lowest variability and standard deviation. Although the survival time of animals in group SM was a little shorter, more than 9 hours, there was no statistical difference among them; in other words, they had similar behavior to that of groups with the spleen, or part of it. On the other hand, animals in group SM recorded survival time of 3.8 hours, which is significantly shorter than that recorded for the other groups. Yet, its variability and standard deviation were high and much higher than those of the other groups. Thus, the survival rate of animals within 10 hours after peritonitis induction was close to 10% in the TS group; 40%, in the SM group; and 72%, in the IP group. This finding is in compliance with the literature, according to which, the remaining splenic tissues after subtotal splenectomy or autoimplantation are capable of keeping the spleen's immunological function, or at least part of it, whenever it is necessary removing the spleen, or part of it^30^ ^-^ ^32^.

The fact that animals subjected to total splenectomy had recorded shorter survival time was expected to happen, since total spleen removal implies the loss of an organ that has essential immunological function. Besides, animals subjected to peritonitis present depressed immunological defense mechanisms, which makes them more susceptible to sepsis and to earlier death. The devastating infection prevails in splenic patients and leads to high mortality rate^33^.

The immunological function of the animals was assessed by counting the subpopulation of TCD4 and TCD8 lymphocytes, since this exam is capable of identifying the subtypes of cells associated with immunity. The ability to measure multiple parameters at the same time in a single cell is likely the most powerful aspect of this technique^34^ ^,^ ^35^.

It was expected that the preservation of the whole spleen (SM) would make it possible prolonging the lives of animals in this group in comparison to animals in the other groups. However, it did not come true, although results recorded for the two surgery groups were similar. Thus, it is possible stating that subtotal splenectomy with the preservation of the inferior pole was able to preserve the animals’ immunological function.

According to the current study, the comparison of TCD4 and TCD8 lymphocyte counting, before and after the spleen procedures, showed that both surgical modalities applied to preserve the splenic tissue (groups 1 and 3) led to similar results, since they presented the same behavior at lymphocyte counting. Animals in these two groups had significant reduction in the number of TCD8 lymphocytes in comparison to group 2 (TS); however, with regards to TCD4 lymphocyte counting, there was no significant difference among the three groups.

A plausible explanation for such an outcome lies on the reduction in the population of TCD8 lymphocytes, assumingly due to surgical stress and/or to blood collection; however, there was no correlation to the assessed surgical procedures, themselves. Based on the experiments with rats, it was possible assessing the effects of the stress caused by animal contention on the pool of blood lymphocytes and on their migration to the skin and mucosa. There was neutrophilia and drop in the number of lymphocytes B, monocytes, NK cells and lymphocytes T right in the first minutes after contention. Nevertheless, after the stress was over, there was fast reversion in the change observed in the number of leucocytes^36^ ^,^ ^37^. Moderate levels of cortisol release during acute stress have induced the redistribution of lymphocytes, they displaced from the blood to tissues more susceptible to infections^38^ ^,39^. They also induce displacement in the auxiliary lymphocytes T (TCD4), from Th1 to Th2. This process makes available an environment rich in anti-inflammatory cytokines, B lymphocytes and eosinophils, which stops the production of inflammatory cytokines and TCD8 cells^40^.

There was no correlation between the survival of animals in the three groups subjected to fecal peritonitis induction and the population of TCD4 and TCD8 lymphocytes. Animals in groups SM and IP were expected to present correlation to TCD4 and TCD8 lymphocyte counting, since they presented longer survival time.

According to the literature, it would be expected that the number of leucocytes in the blood, mainly of lymphocytes, would increase after splenectomy^8^ ^,^ ^9^; however, the current results did not evidence any increase in the number of lymphocytes in the blood. Such an outcome must have resulted from the time the second blood collection was carried out, i.e., 70 days after the splenectomy. Research design took into account that the splenic tissue could recover its immunological function after this time, when any spleen surgery type is performed. Unfortunately, it may have happened some sort of accommodation in the level of these lymphocytes in the blood within this period-of-time, because this organ had its function recovered 8 weeks after the spleen procedures, either after manipulation or subtotal splenectomy with the preservation of the inferior pole.

It would be interesting conducting further studies about peritonitis in animals with their spleen, or with part of it, in order to assess their survival time rates. Studies conducted with experimental animals have shown that local anesthetics such as 0.5% bupivacaine or 2% lidocaine were effective in avoiding death in 100% of animals with fecal perotinitis^26^.

We believe that the present study has contributed to improve the knowledge about the importance of spleen against infection cases. However, new investigations in this topic should include a control group (without any procedure) in order to rule out possible biases observed in the current study.

## Conclusions

Based on the present results, the maintenance of the splenic tissue helps prolonging the survival time of rats. There was change in the number of TCD8 lymphocytes but there was no correlation between survival rate and the number of TCD4 and TCD8 lymphocytes.
